# Oral breathing and its dental and cognitive implications: a structured narrative review with a pediatric focus

**DOI:** 10.3389/fdmed.2026.1868240

**Published:** 2026-10-27

**Authors:** Alex Towery, Gopikrishna Deshpande

**Affiliations:** 1 Biomedical Science Program, College of Sciences and Mathematics, Auburn University, Auburn, AL, United States; 2 Auburn University Neuroimaging Center, Department of Electrical and Computer Engineering, Auburn University, Auburn, AL, United States; 3 Department of Psychological Sciences, Auburn University, Auburn, AL, United States; 4 Alabama Advanced Imaging Consortium, Birmingham, AL, United States; 5 Center for Neuroscience, Auburn University, Auburn, AL, United States; 6 Department of Psychiatry, National Institute of Mental Health and Neurosciences, Bangalore, India; 7 Department of Heritage Science and Technology, Indian Institute of Technology, Hyderabad, India

**Keywords:** cognitive development, craniofacial development, mouth breathing, nasal nitric oxide, pediatric dental development

## Abstract

Oral breathing is a common condition, particularly in pediatric populations, that has been independently associated with dental abnormalities and cognitive dysfunction. However, these outcomes are often studied separately, limiting a comprehensive understanding of their potential interconnection. This structured narrative review synthesizes current literature to examine both the dental and cognitive consequences of habitual oral breathing and to highlight their possible shared physiological mechanisms, including a hypothesized role of disrupted nasal nitric oxide signaling. A structured PubMed search was conducted in October 2024 using two targeted strategies: one focused on dentofacial health outcomes and another on neurological and cognitive effects. Twenty-three primary studies were included in the evidence synthesis (fourteen dental and nine cognitive), and five reviews and editorials were consulted as contextual background. Across multiple studies, oral breathing is associated with malocclusion, differences in craniofacial morphology, and increased risk of oral disease. Concurrently, research suggests associations with reduced working memory, attention difficulties, altered brain oxygenation patterns, sleep-disordered breathing, and decreased academic performance. By setting these findings side by side, this review proposes a hypothesis-generating framework in which oral breathing is considered a possible marker of broad developmental and physiological risk; no included study examined dental and cognitive outcomes in the same participants. These findings emphasize the importance of early interdisciplinary identification and intervention, particularly within pediatric dental care, and highlight the need for future research exploring causal pathways between respiratory patterns, oral health, and cognitive development. Findings should be interpreted in light of the review's limitations, including reliance on a single database for the primary searches, heterogeneity in the diagnostic criteria for oral breathing, the predominance of observational designs, the absence of a formal risk-of-bias assessment, and reliance on adult experimental studies for several cognitive findings.

## Introduction

Oral breathing is a common oral habit observed most often in children that has been associated with a range of adverse health outcomes. It is defined as air passing through the mouth instead of the nose and is primarily caused by some upper airway obstruction which reduces nasal airflow and causes air to enter completely or partially through the oral cavity. Oral breathing has been associated with several negative oral health issues, including dental health, maxillofacial health, and cranial development. There is also evidence associating oral breathing with differences in cognitive and behavioral measures, although this evidence base is smaller and more heterogeneous.

The majority of studies reviewed focused on oral breathing in children. Oral breathing is more often observed in children than adults and it is one of the most common deleterious oral habits in children. It is almost always caused by an upper airway obstruction which forces the child to habitually breathe through their mouth. The most common cause of this obstruction is adenotonsillar hypertrophy (enlarged adenoids and/or tonsils) (1). Nasal septum deviation can also cause obstruction of airflow (2). Another common cause of chronic oral breathing among children is allergic rhinitis (hay fever) (3). This review will not focus on the specific causes of oral breathing, but rather will bring attention to the dental and cognitive health consequences that often accompany this habit.

It is important to distinguish habitual oral breathing from the underlying conditions that most often cause it. Adenotonsillar hypertrophy, allergic rhinitis, and other forms of upper airway obstruction are the most common causes of chronic oral breathing in children, and each can independently affect craniofacial development, sleep, and neurocognitive function. Outcomes observed in mouth-breathing children may therefore reflect the breathing pattern itself, the underlying obstructive condition, or both.

Oral breathing may plausibly play three non-mutually-exclusive roles: as a contributing cause of downstream outcomes, for example through altered orofacial muscle balance or loss of nasal physiological functions; as a mediator on the pathway between upper airway obstruction and adverse outcomes; and as a readily observable clinical marker of underlying airway pathology. The observational designs that dominate this literature cannot distinguish between these roles. This review therefore treats oral breathing primarily as a clinically detectable marker warranting further evaluation, and discusses hypothesized causal and mediating pathways as hypotheses.

The first main purpose of this review is to investigate the correlations between habitual oral breathing and the many negative physiological effects it can bring, including altered craniofacial development, dental malocclusions, and increased susceptibility to oral diseases. These effects can significantly impact both the appearance and function of the teeth, jaws, and surrounding structures. By synthesizing current research, this review aims to provide a clearer understanding of these physiological outcomes and emphasize the importance of early diagnosis and intervention to prevent or mitigate these harmful effects. Furthermore, exploring these correlations can highlight the need for improved treatment strategies and preventative measures in pediatric care, ensuring that oral breathing is addressed before it leads to long-term health problems.

The other main purpose of this review is to investigate the correlations between oral breathing and cognitive impairment. While much of the research on oral breathing has focused on its impact on dental and craniofacial development, emerging studies suggest that it also has significant neurological consequences. The brain relies on efficient oxygenation and proper respiratory patterns to function optimally, and disruptions caused by habitual oral breathing may contribute to cognitive deficits. Research has begun to establish associations between oral breathing and impairments in memory, attention, and executive function, with potential long-term consequences for learning and academic performance. By exploring these connections, this review seeks to shed light on the cognitive risks posed by oral breathing and encourage further research in this area.

Previous reviews have examined the effects of oral breathing on either dentofacial development or specific aspects of neurological function. For example, Lin et al. (1) provided a concise review of mouth breathing's impact on dentofacial development, while several neuroimaging studies have explored cognitive effects independently. Jefferson (4) published an early review addressing facial growth, health, academics, and behavior together, establishing a foundation for integrated thinking about oral breathing's consequences. More recently, Jung and Kang (5) explicitly noted the gap between dental health research and cognitive research in oral breathing, calling for studies that collect dental health indicators alongside brain activity data. Masutomi et al. (6) similarly framed dental-functional and cognitive outcomes as part of the same clinical problem. Despite these contributions, there remains limited synthesis that systematically examines both dental and cognitive outcomes side by side and proposes shared physiological mechanisms. This review aims to address this gap by integrating evidence from both domains to provide a more comprehensive understanding of oral breathing as a condition with both structural and functional implications.

The overarching goal of this review is to examine the dental and cognitive literatures on oral breathing side by side and to formulate testable hypotheses about mechanisms that could link them. To explore these interconnected outcomes, this paper presents a structured narrative review using a transparent PubMed search strategy. By synthesizing findings from both dental and neurological literature, this review aims to provide a more integrated understanding of how oral breathing affects overall health and to emphasize the importance of interdisciplinary approaches in both diagnosis and treatment.

## Methods

Two structured Boolean searches (Search #1, dental outcomes; Search #2, cognitive and neurological outcomes) and one supplementary cross-domain search were run in PubMed in October 2024. All searches used the complete strings reported below, across all fields, with no date, language, species, or publication-type filters; language, study design, population, and outcome criteria were applied at screening. To reproduce the retrieval as run, each string can be combined with the entry-date limit AND (“1800/01/01”[Date - Entry] : “2024/10/11”[Date - Entry]); with this limit, Search #1 returns 246 records, Search #2 returns 117 records, and the cross-domain search returns 92 records. The source of each included study is listed in Supplementary Table S1. Consistent with the format of a structured narrative review, findings and their interpretation are presented in an integrated discussion section following the summary of evidence.

Titles and abstracts of all records retrieved by each search were screened against the inclusion and exclusion criteria by the first author (A.T.). Full texts of potentially eligible records were then assessed for eligibility, and the resulting selections were reviewed and verified by the senior author (G.D.). Disagreements were resolved by discussion until consensus was reached. Reasons for full-text exclusion are reported in Figures 1, 2.

**Figure 1:**
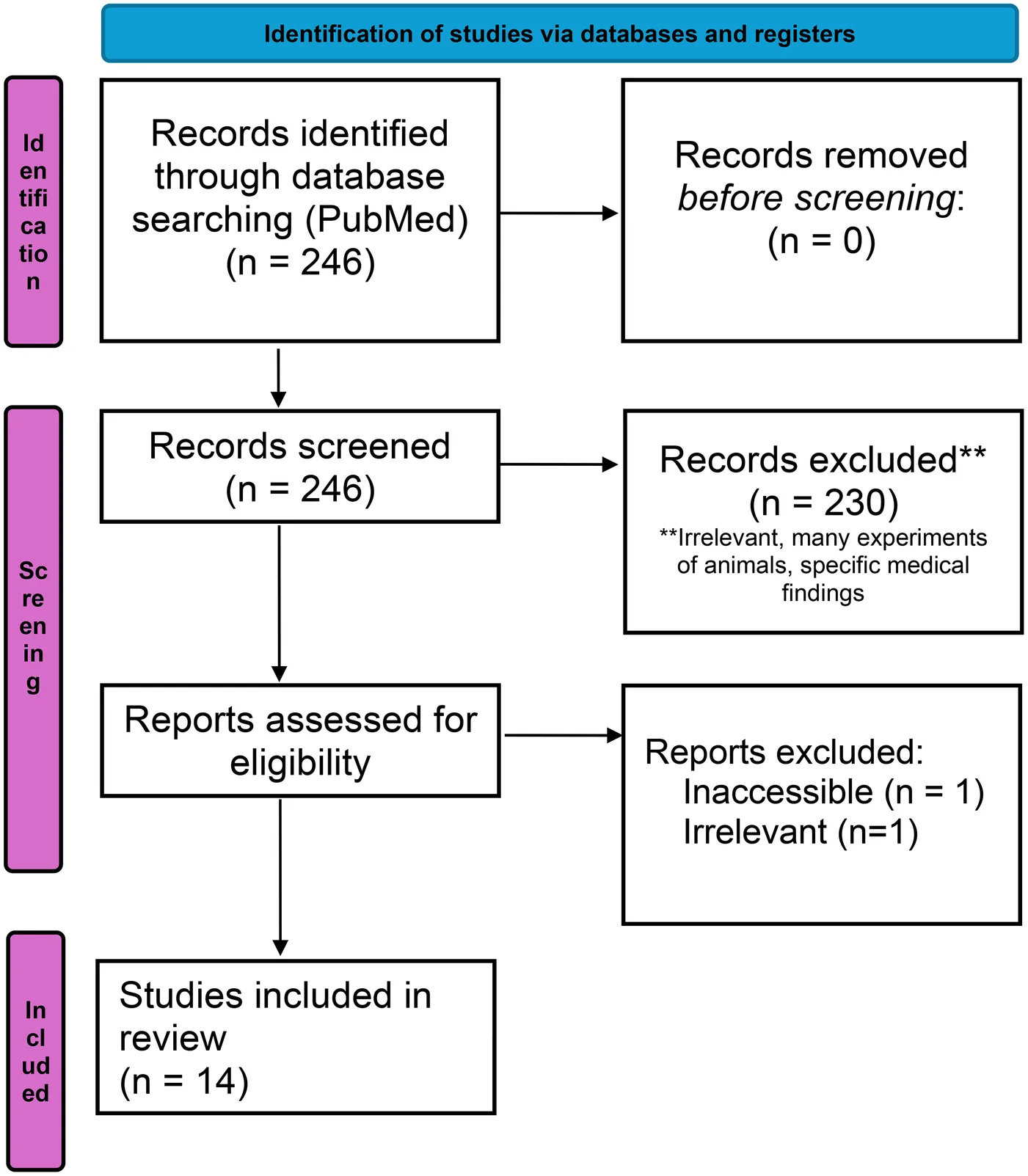
Selection process for studies in the first search, i.e., correlations between dental problems and breathing patterns.

**Figure 2:**
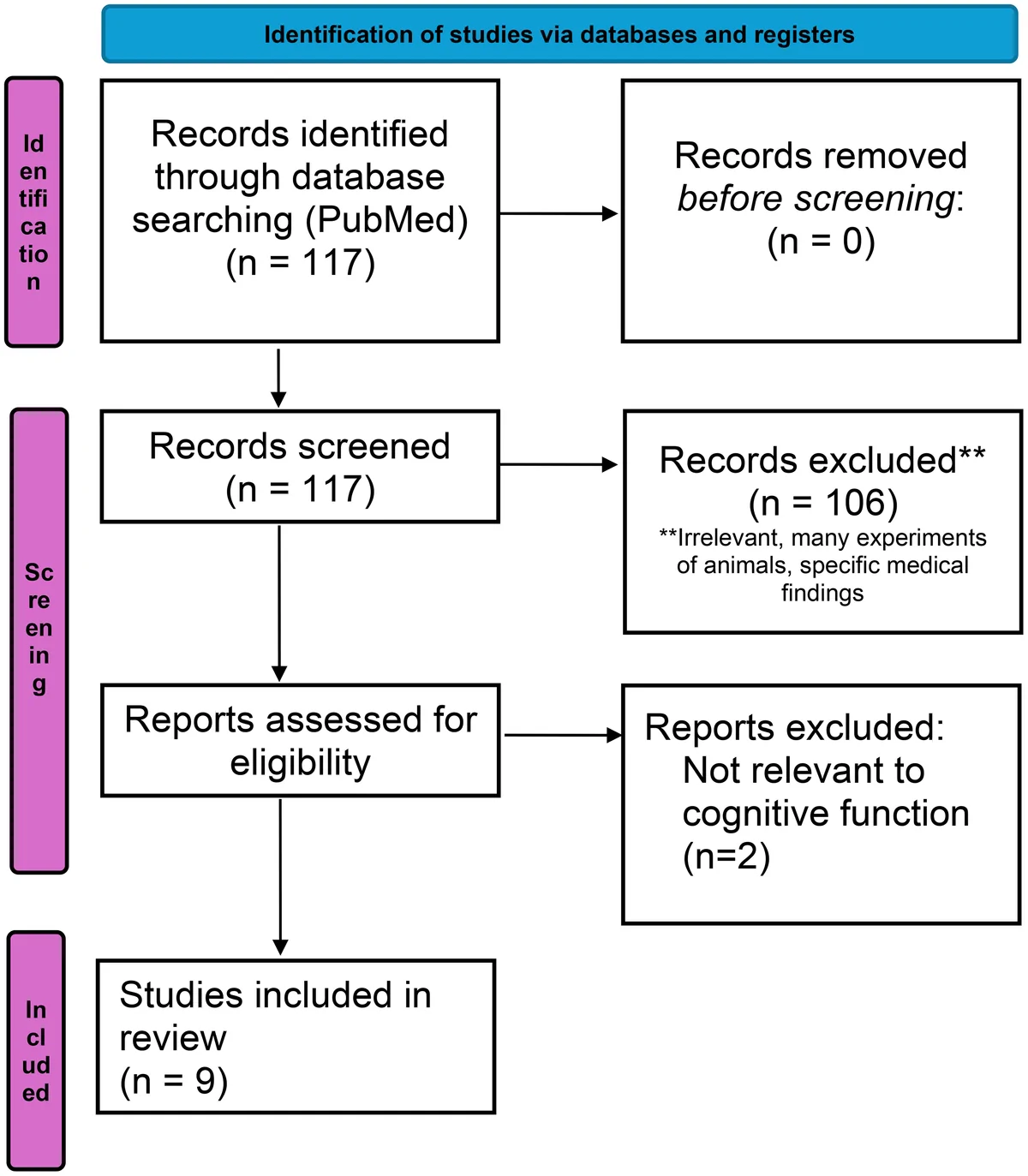
Selection process for studies in the second search, i.e., correlations between breathing patterns and cognitive function.

### Inclusion and exclusion criteria

Studies were included if they: (1) were published in English in peer-reviewed journals; (2) investigated the effects or associations of oral or mouth breathing on dental, craniofacial, or cognitive outcomes; (3) involved human participants, with preference for pediatric populations; and (4) were original research studies, including cross-sectional, cohort, case-control, and experimental designs. Studies were excluded if they: (1) were conducted exclusively on animal models; (2) focused on surgical or pharmacological interventions without reporting breathing-related outcomes; (3) were conference abstracts, editorials, or letters without original data; or (4) were not accessible in full text. Review articles were excluded from the primary evidence synthesis but were consulted for contextual background.

### Use of adult experimental evidence

Controlled experimental manipulation of breathing route combined with neuroimaging (EEG, NIRS, fMRI) has to date been performed almost exclusively in adults, as such protocols are difficult to implement in children. Adult experimental studies were therefore included as mechanistic, hypothesis-generating evidence regarding the acute effects of breathing route on brain function, and not as evidence of developmental consequences in children. These studies are labeled as adult-derived at each point of citation, are flagged in Table 1, and are interpreted with the explicit caveat that acute effects in adults may not reflect the neurodevelopmental consequences of chronic oral breathing during childhood.

**Table 1:** Characteristics of the 23 primary empirical studies included in the evidence synthesis.

Ref	Authors, year	Design	Population (age)	*n*	OB diagnosis	Key outcomes	OCEBM	Key limitations	Domain
(2)	Farid & Metwalli, 2010	CT cross-sect.	Children, 10–15 y	67	CT imaging	Airway obstruction, adenoid hypertrophy, OSA risk	4	Single center; imaging-based grouping; no functional outcomes	Dental
(3)	Alhazmi, 2022	Cross-sect.	Children/adol., 9–17 y	498	Clinical exam	Speech disorders, malocclusion types	4	Mouth breathers only; no nasal-breathing comparison group	Dental
(6)	Masutomi et al., 2024	Cross-sect.	Adol./young adults, 12–25 y	103	Questionnaire + exam	Reduced lip-closing force, tongue pressure, chewing efficiency	4	Single clinic; broad age span; no cognitive outcomes	Dental
(8)	Grippaudo et al., 2016	Cross-sect.	Children, 7–13 y	3,017	Clinical exam (ROMA)	Malocclusion associated with oral habits and MB	4	Cross-sectional; no MB severity grading	Dental
(9)	Jaiswal et al., 2023	Ceph. cross-sect.	Adults/adol.	40	Clinical + ceph.	Reduced SNA, airway changes; head posture NS	4	Small sample; head-posture difference not significant	Dental
(10)	Lee et al., 2021	Cross-sect.	Children, 8–11 y	1,507	Parent report	Atopic dermatitis association; no caries association	4	Parent-reported MB; caries association absent	Dental
(11)	Paolantonio et al., 2019	Cross-sect.	Preschoolers, 3–6 y	1,616	Clinical exam	Open bite, overjet, crossbite	4	Cross-sectional; preschool dentition only	Dental
(13)	Lysy et al., 2021	Ceph. retrospective	Children (age NR)	123	Clinical exam	Decreased maxillary intermolar width	4	Retrospective; age range not reported	Dental
(14)	Nagaiwa et al., 2016	Experimental	Adults^a^	10	Instructed breathing	Decreased chewing efficiency during MB	3	Very small sample; acute manipulation; adults	Dental
(15)	Wang et al., 2023	Retrospective	Children, 7–12 y	126	Clinical exam	Decreased cortical bone density and width	4	Retrospective; exploratory screening model	Dental
(16)	Bakor et al., 2010	Prospective single-group	Children, 9–13 y	20	Clinical exam	Tooth demineralization during maxillary expansion	4	No control group; small sample	Dental
(17)	Kaur et al., 2018	Controlled clinical study	Adults^a^ (periodontitis)	66	Clinical exam	Impaired scaling/root-planing outcomes in MBs	3	Adult periodontitis patients; generalizability to children unclear	Dental
(18)	Alqutami et al., 2019	Cross-sect.	Children, 10–15 y	785	Questionnaire + exam	Halitosis association; caries and gingivitis NS	4	Self-reported MB; two index teeth only	Dental
(19)	Jiménez et al., 2017	Case-control	Children (age NR)	1,204	Clinical exam	Lower blood O2, more infections, malocclusions	4	Age not reported in abstract; regional sample	Dental/General
(5)	Jung & Kang, 2021	Experimental fMRI	Adults^a^, mean 22 y	22	Controlled voluntary breathing	Reduced working-memory-related connectivity during oral breathing	3	Acute manipulation; healthy adults; no dental outcomes	Cognitive
(21)	Arshamian et al., 2018	Experimental	Adults^a^, 19–25 y	24	Nose clip/mouth tape	Better odor-memory consolidation with nasal breathing	3	Odor memory only; acute; small sample	Cognitive
(22)	Lee et al., 2020	Experimental EEG	Adults^a^	20	Controlled	Reduced EEG power during MB; task performance NS	3	Behavioral null result; acute; small sample	Cognitive
(23)	Kuroishi et al., 2015	Cross-sect.	Children, 7–10 y	55	Clinical + ENT exam	Lower reading, arithmetic, pseudoword working memory; number working memory NS	4	Unbalanced groups (42 vs 13); no IQ measure; unadjusted analyses	Cognitive
(24)	Hong et al., 2021	Experimental EEG	Adults^a^	20	Controlled + O2 supplement	EEG classification of breathing patterns	3	Oxygenation not directly measured; acute	Cognitive
(25)	Sano et al., 2013	Experimental NIRS	Adults^a^	9	Controlled	Increased prefrontal oxygen exchange (angle k) and deoxy-Hb	3	*n* = 9; acute; voluntary-effort confound	Cognitive
(26)	Yoshimura et al., 2019	Experimental (2 exps.)	Adults^a^, 18–28 y	15 + 36	Nasal plug/mouth tape	Slower difficult visual search (Exp. 2); Exp. 1 null	3	Exp. 1 null result; university students; acute	Cognitive
(27)	Jung et al., 2020	Experimental rs-fMRI	Adults^a^	21	Controlled	Functional connectivity differences between breathing routes	3	Acute; exploratory interpretation	Cognitive
(34)	de Braga Junior et al., 2020	Cross-sect.	Children, 7–11 y	30	Clinical classification	Poorer sustained auditory attention (inattention, total errors)	4	Small sample; identified via supplementary targeted search	Cognitive

OB, oral breathing; MB, mouth breathing; Cross-sect., cross-sectional; Ceph., cephalometric; Adol., adolescents; NS, not statistically significant; NR, not reported in the source; OCEBM, Oxford Centre for Evidence-Based Medicine 2011 study-design level (a design hierarchy, not a risk-of-bias assessment); OSA, obstructive sleep apnea.

a Adult study: included as acute mechanistic evidence only.

### Search #1: correlations between dental problems and breathing patterns

The first search strategy aimed to identify dental problems that directly link to oral breathing. The goal of the first PubMed search was to find papers that discussed oral breathing and its correlation to dental-related issues while excluding papers that focused on cognitive activity. To find papers discussing oral breathing and its comparisons to nasal breathing, the following was included: (“oral breathing” OR “nasal breathing” OR “mouth breathing” OR “oral respiration” OR “nasal respiration” OR “mouth respiration”). To ensure the paper also discussed oral breathing's implications on dental health the following was added: AND (“dental” OR “malocclusion”).

The first search, (“oral breathing” OR “nasal breathing” OR “mouth breathing” OR “oral respiration” OR “nasal respiration” OR “mouth respiration”) AND (“dental” OR “malocclusion”) yielded 982 results. Most of these were irrelevant to the study, and many were too involved in malocclusion specifically to be useful. For this reason, the alternate search excluded “malocclusion” to help narrow down the search. Many papers also did not discuss the direct impacts/effects of oral breathing on dental health, so for this reason the following was added: AND (“impact” OR “effect” OR “association” OR “cause” OR “influence”). Finally, some papers discussed oral breathing's effects on the brain, which needed to be reserved for the next search. To differentiate these papers from ones discussing brain cognition, the following was added: NOT (“brain” OR “cognitive” OR “cognition” OR “neurological”).

### Search #2: correlations between breathing patterns and cognitive function

The second step was to find papers discussing oral breathing and its connection to cognitive activity, while excluding papers focused on dental issues. Using what was learned from the first PubMed search, this search was thought of as a simple reversal of the first search. For this search, the original (“oral breathing” OR “nasal breathing” OR “mouth breathing” OR “oral respiration” OR “nasal respiration” OR “mouth respiration”) remained to keep the focus on oral breathing. This time key words related to the brain, (“brain” OR “cognitive” OR “cognition” OR “neurological”), were added, not excluded. “Dental” and “malocclusion” needed to be excluded, due to the high volume of papers related to these topics in the first search.

### Supplementary cross-domain search

Because Searches #1 and #2 used mutual exclusion terms, studies reporting both dental and cognitive outcomes could have been excluded by both. A supplementary PubMed search was therefore run in October 2024 using (“mouth breathing” OR “oral breathing”) AND (“dental” OR “malocclusion” OR “facial growth”) AND (“cognitive” OR “academic” OR “behavior” OR “attention”), without exclusion terms or filters. It retrieved 92 records, which were screened against the same eligibility criteria and yielded four additional records (4–7): a narrative review (4) and an editorial (7), used as contextual background only, and two primary studies that each measured outcomes in a single domain, Jung and Kang (5) (cognitive) and Masutomi et al. (6) (dental). No study included in the synthesis measured both dental and cognitive outcomes in the same participants.

### Database choice and supplementary coverage search

PubMed/MEDLINE was used for the primary searches because the clinical literature relevant to this review — pediatric dentistry, orthodontics, otolaryngology, sleep medicine, and cognitive neuroimaging — is predominantly published in MEDLINE-indexed journals, and because the PubMed interface supports fully reproducible Boolean search strings, consistent with the transparency goals of a structured narrative review. Databases such as Scopus, Embase, Web of Science, and PsycINFO index additional titles, particularly in the psychological sciences and in regional journals, and their omission may mean that relevant studies were not retrieved. During the first revision, on 27 August 2026, a supplementary search was run in Semantic Scholar (Academic Graph API, bulk paper search), which indexes journals not covered by MEDLINE. Two queries paralleling the primary searches were used, with no filters: dental, (“mouth breathing” | “oral breathing” | “mouth breathers” | “mouth breather” | “oral respiration” | “mouth respiration”) + (malocclusion | dental | craniofacial | orthodontic | “facial growth” | occlusion), which retrieved 1,517 records; and cognitive, the same breathing terms + (brain | cognitive | cognition | neurological | memory | attention | academic | neurocognitive), which retrieved 426 records. Records published from 1990 onward were filtered by title for a breathing-route term together with an outcome term, and records already included in the review were removed. Because the purpose of this search was to identify eligible studies not indexed in PubMed, the dental filter was applied only to records without a PubMed identifier (101 records), whereas the cognitive filter was applied to all records (16 records). These titles were assessed against the eligibility criteria, the abstract and full text were retrieved where eligibility could not be decided from the title, and each candidate was checked against PubMed. One eligible study without a PubMed record was identified and included: de Braga Junior et al. (34), published in Revista CEFAC. Semantic Scholar results reflect its index on the search date. This supplementary search was not a systematic multi-database search and does not establish complete coverage of the literature; reliance on PubMed for the primary searches is retained as a limitation.

### Study design classification

Study-design level was classified using the 2011 Oxford Centre for Evidence-Based Medicine (OCEBM) framework and is reported in Table 1 alongside a study-specific limitations column. The OCEBM framework is a hierarchy of study designs; it is not a judgment of methodological quality and not a risk-of-bias assessment. A formal risk-of-bias assessment was not performed. This review does not pool effect estimates, and the included studies span cross-sectional, case-control, retrospective, single-group prospective, controlled clinical, and acute crossover experimental designs, with exposures ranging from clinically diagnosed habitual oral breathing to reported and instructed mouth breathing; appraisal would therefore have required several design-specific instruments whose ratings could not be compared across the evidence base. The SANRA scale for narrative review articles includes the presentation of evidence level among its quality items (35), which the design-level classification addresses. The absence of a formal risk-of-bias appraisal nonetheless limits the certainty of the conclusions, as discussed under Certainty of the Conclusions and in the Limitations.

## Results

### Search #1 results

The finalized first search, (“oral breathing” OR “nasal breathing” OR “mouth breathing” OR “oral respiration” OR “nasal respiration” OR “mouth respiration”) AND (“dental”) AND (“impact” OR “effect” OR “association” OR “cause” OR “influence”) NOT (“brain” OR “cognitive” OR “cognition” OR “neurological”), yielded 246 results. This result was more manageable and led to 16 results that all correlate a variety of dental, dentofacial, and cranial health issues to oral breathing. Upon further screening against inclusion criteria, 2 of the 16 studies were excluded (one inaccessible, one irrelevant), resulting in 14 studies included to examine the relationship between oral breathing and dental outcomes (Figure 1).

### Search #2 results

The finalized second search, (“oral breathing” OR “nasal breathing” OR “mouth breathing” OR “oral respiration” OR “nasal respiration” OR “mouth respiration”) AND (“brain” OR “cognitive” OR “cognition” OR “neurological”) NOT (“dental” OR “malocclusion”) yielded 117 results. Of this, 11 appeared to be useful and relevant to oral breathing's effects on the brain and cognitive ability. Upon further screening, 2 of the 11 studies were excluded due to lack of relevance, resulting in 9 studies included to examine the relationship between oral breathing and cognitive outcomes (Figure 2). Four additional records were identified through the cross-domain search and classified as described in the Methods.

### Summary of included studies

The two primary searches and the supplementary cross-domain search together yielded 27 included records. Five of these are reviews or editorials and are used as contextual background only (Table 2); the remaining 22 are primary empirical studies. One additional primary study identified through supplementary targeted searching during revision was added (34), giving 23 primary studies in the evidence synthesis (14 dental and 9 cognitive). Table 1 presents these studies, organized by domain, with sample size, age range, study-design level (OCEBM 2011), and key limitations; adult studies are flagged with an asterisk and are interpreted as acute mechanistic evidence only. Table 2 lists the contextual sources, which are not part of the evidence synthesis and are not assigned design levels.

**Table 2:** Reviews and editorials consulted for contextual background. These sources are not part of the primary evidence synthesis and are not assigned study-design levels.

Ref	Authors, year	Type	Topic	Role in review
(1)	Lin et al., 2022	Narrative review	Mouth breathing and dentofacial development	Contextual background (Introduction; dental framing)
(4)	Jefferson, 2010	Narrative review/case reports	Facial growth, health, academics, behavior	Contextual background (Introduction; sleep and behavior discussion)
(7)	Tamkin, 2020	Editorial	Airway dysfunction and dental health	Contextual background (nitric oxide physiology framing)
(12)	Huynh et al., 2014	Narrative review	Sleep disorders and oral disease interactions	Contextual background (sleep–oral disease discussion)
(20)	Shahsavar et al., 2024	Narrative review	Nasal respiration and brain dynamics	Contextual background (cognitive mechanisms)

## Summary of evidence

### Dental and craniofacial findings

#### Malocclusion

The most prominent association to oral breathing of any health complication found is malocclusion, particularly in children. It is well established that malocclusion appears more frequently in mouth-breathing children than in nasal-breathing children (1). Multiple cross-sectional studies (8–10), summarized also in a narrative review (1), note a higher prevalence of Class II malocclusion (overbite) in oral breathers compared to the general population. Though Class II malocclusion is the most observed trend among oral breathers, open bite and posterior crossbite have also been associated (3, 11). A cross-sectional study of 1,616 Italian preschoolers aged 3–6 found a statistically significant association connecting oral breathing to anterior open bite, increased overjet (buck teeth), posterior crossbite, and displacement (11). Class III malocclusion (underbite) has also been reported in association with oral breathing (1, 12), though direct evidence is more limited than for Class II. Oral breathers are also known to have a higher prevalence of narrow dental arches and dental crowding, notably on the upper arch (8).

#### Craniofacial development

In addition to malocclusion, overall craniofacial development is also strongly correlated to oral breathing. Children with mouth breathing often present features including maxillary (upper jaw) protrusion, mandibular (lower jaw) retrusion, clockwise rotation of the mandible, protruding upper lip, and incompetent lip seal (1). Typical facial features among mouth breathers also include a “long face,” contracted upper dental arch, high arched palate, and gummy smile (8, 11). Jefferson (4) described these collectively as part of “adenoid facies” and emphasized the importance of screening from as early as age five. A cephalometric cross-sectional study comparing 20 mouth breathers to 20 nasal breathers found statistically significant differences in cranial morphology among mouth breathers including a reduced SNA (Sella-nasion-point A angle), a reduced maxilla length, an increased gonial angle, an increased palatal-mandibular plane angle, and a decreased upper and lower pharyngeal width when compared to nasal breathers (9). A retrospective cephalometric study of 123 children found a significant correlation between oral breathing and a decreased maxillary intermolar width (13). A systematic review and meta-analysis of ten comparable studies likewise found significantly reduced SNA and SNB angles and an increased ANB angle in mouth-breathing children; the certainty of this evidence, assessed by the authors using GRADE, was rated low to moderate (28). In an acute experimental study of ten adults, masticatory efficiency (chewing activity strength) was decreased during mouth breathing (14). Consistent with these findings, a retrospective study of 126 children aged 7–12 found that oral breathing was associated with a decreased trend of cortical bone mineral density and cortical bone width (15).

#### Oral and general health

The effects of oral breathing on dental health are clinically significant. Normal nasal breathing allows saliva in the oral environment to stabilize pH, digest food, combat bacteria, and prevent dental caries (cavities). In oral breathers, airflow in the mouth causes saliva to evaporate and decreases humidity in the oral cavity, leading to higher risk of caries, higher levels of streptococcus mutans and higher levels of plaque (1, 16). Individuals with xerostomia (dryness of the mouth) tend to have decreased epithelial cells and a dehydrated gingival surface that can contribute to gingivitis, periodontitis and other periodontal diseases, as reported in adults with chronic periodontitis (17). A cross-sectional study of 785 children aged 10–15 found a significant association between mouth breathing and halitosis (bad breath), with no statistically significant differences in caries or gingival indices between mouth and nasal breathers (18).

Oral breathing has implications on the overall health of children as well. Improper airway flow can lead to an overall decrease in oxygen intake. A case-control study of 1,204 children found a lower blood oxygen concentration among oral breathers than among controls. They had a higher prevalence of colds, ear infections, and tonsillitis than nasal breathers within the previous year. Higher rates of allergies were observed in oral breathers than nasal breathers (19). Oral breathing may thus have implications for general health and immune function in children, although these observations derive from cross-sectional comparisons.

### Cognitive and neurological findings

The cognitive evidence base is considerably smaller and more heterogeneous than the dental literature: nine primary studies compared with fourteen, spanning EEG, NIRS, fMRI, and behavioral assessment methodologies, with the majority of the experimental work conducted in adults and only two studies conducted in children. No study included in the synthesis assessed dental and cognitive outcomes in the same participants; any link between the two domains therefore remains a hypothesis. Conclusions in the cognitive domain should therefore be regarded as substantially more preliminary than those concerning dentofacial outcomes.

To evaluate the effects of breathing patterns on cognitive function, the studies included in this review utilized a range of neuroimaging and cognitive assessment tools. Electroencephalography (EEG) measures electrical activity in the brain and is commonly used to assess brain wave patterns associated with attention and working memory. Near-infrared spectroscopy (NIRS) is a non-invasive method that measures oxygen levels in specific regions of the brain, providing insight into brain activity and oxygen distribution during different breathing conditions. Functional magnetic resonance imaging (fMRI) maps brain activity by detecting changes in blood oxygenation. In addition, many studies used standardized cognitive assessments to evaluate functions such as memory, attention, language comprehension, and executive functioning. Together, these methods allow researchers to examine both the physiological and functional effects of oral breathing on the brain.

Building on these methods, evidence suggests that nasal breathing is optimal for healthy brain activity. A narrative review describes how nasal airflow activates olfactory sensory neurons in the nasal cavity, entraining brain activity across regions involved in cognition and memory (20). An acute experimental study in healthy adults found that nasal respiration during a consolidation period enhanced recognition of odor memories compared with oral respiration (21). Across the studies comparing breathing routes, the balance of evidence suggests an association between oral breathing and less favorable indices of brain function, although several studies also report null findings on behavioral measures, as noted below.

#### Memory

Studies in both adults and children have reported working-memory-related differences associated with breathing route (22, 23), while an acute experimental study in 24 healthy adults found that nasal breathing during a one-hour consolidation period enhanced recognition of episodic odor memory compared with oral breathing (21); whether this effect extends to other memory domains has not been established. Working memory is the cognitive system responsible for temporary storage and information processing. An acute experimental EEG study in 20 adults observed reduced power in frequency bands linked to working memory during oral breathing, although task accuracy and response times did not differ significantly between breathing routes (22). A cross-sectional study of 55 children aged 7–10 found lower scores among mouth breathers in reading comprehension, arithmetic, and phonological working memory for pseudowords, with no significant group difference in working memory for numbers (23). In an acute fMRI experiment in 22 healthy adults, Jung and Kang (5) found that functional connectivity in regions associated with working memory, including the left cerebellum and bilateral inferior parietal gyrus, was significantly reduced during oral breathing compared to nasal breathing.

#### Oxygen in the brain

The relationship between breathing route and cerebral oxygenation is more complex than early reports suggested, and the available measures capture different physiological constructs. In the case-control study of children described above, oral breathers had lower blood oxygen concentration than controls (19), and mouth-breathing children in a cross-sectional study demonstrated greater difficulty with working memory and arithmetic skills, which the study's authors attributed in part to daytime tiredness and loss of attention (23).

Sano et al. (25) used vector-based near-infrared spectroscopy in nine healthy adults and found that the angle k, an index derived from changes in oxyhemoglobin and deoxyhemoglobin, was significantly greater during voluntary mouth breathing. Mouth breathing was accompanied by increased deoxyhemoglobin without a significant increase in oxyhemoglobin. These findings indicate altered local prefrontal hemodynamics and oxygen exchange during acute mouth breathing, but they do not demonstrate excess cerebral oxygen, reduced global cerebral oxygenation, or reduced systemic oxygen delivery. The result may reflect changes in local oxygen extraction, neural or respiratory effort, or other hemodynamic factors; the authors themselves note that it may be no more than a consequence of the voluntary effort required to breathe through the nonpreferred route. Given the small adult sample and the acute voluntary manipulation, its relevance to chronic oral breathing in children remains uncertain.

Separately, Hong et al. (24) reported EEG differences between nasal breathing, mouth breathing, and mouth breathing with supplemental oxygen during a working-memory task in twenty adults, and interpreted these differences as related to oxygen availability. Because cerebral and systemic oxygenation were not directly quantified in that study, it does not establish that mouth breathing reduces oxygen delivery to the brain. The evidence in this area is therefore best described as showing altered brain activity and local hemodynamics under acute changes in breathing route, with the question of cerebral oxygen delivery unresolved.

#### Sleep disorders

Oral breathing has been associated with sleep disorders in both contextual reviews and primary studies (4, 12). Most notably is obstructive sleep apnea, in which throughout sleep, the muscles supporting the throat, tongue, and soft palate relax and temporarily close off airflow and stop breathing. High incidences of obstructive sleep apnea are reported in mouth breathing adolescents. These adolescents, compared to healthy adolescents, have been reported to show poorer learning ability, more behavioral problems, and worse concentration skills, findings attributed by that review's author to disrupted sleep from oral breathing (4); as a single-source narrative observation, this should be interpreted with caution. Sleep-disordered breathing is associated with intermittent hypoxia. However, a systematic review of the pediatric OSA literature found that although intellectual ability in children with OSA may be impaired, it remains within the normal range, with insufficient evidence for deficits in specific cognitive domains owing to low levels of evidence, conflicting findings, and heterogeneous measures (29). Consistent with the complexity of this relationship, the CHAT randomized trial found that adenotonsillectomy improved behavior, quality of life, symptoms, and polysomnographic findings in children with OSA, but did not significantly improve the primary attention and executive-function outcome (30). Jefferson (4) similarly emphasized that children with mouth breathing do not sleep well due to obstructed airways, and that many are misdiagnosed with attention deficit disorder.

#### Academic and motor skills

The comparison of academic skills between healthy children and children with mouth-breathing syndrome has received sustained attention. The available studies, although few in number and largely cross-sectional, report poorer academic achievement and cognitive test performance in mouth-breathing children than in nasal-breathing children. Kuroishi et al. (23) found lower scores in reading comprehension, language comprehension, phonological working memory, and arithmetic among oral breathers. They attributed this in part to daytime tiredness and loss of attention, which lead to poorer motivation and learning ability. In a cross-sectional study of 30 children aged 7–11 identified through supplementary targeted searching during revision, mouth- and oronasal-breathing children showed more auditory-behavior complaints and poorer sustained auditory attention, with significantly more inattention errors and total errors, than nasal breathers (34). In two acute visual-search experiments in adults (*n* = 15 and *n* = 36), Yoshimura et al. (26) found no effect of breathing route in the first experiment; in the second, participants required more time to find poorly discriminable targets during oral breathing, suggesting an effect on pre-search sensory processing that emerges only under difficult search conditions. In an acute resting-state fMRI experiment in 21 healthy adults, Jung et al. (27) observed more widespread and left-lateralized functional connectivity during oral respiration than during nasal respiration; the functional significance of these differences remains to be established. In a cross-sectional study of 103 adolescents and young adults, Masutomi et al. (6) found significantly reduced lip-closing force, tongue pressure, and masticatory efficiency in mouth breathers, indicating an association between oral breathing and oral-motor function; this study measured no cognitive outcomes.

## Discussion

### A hypothesized role for nitric oxide in linking breathing mode to health outcomes

A hypothesized physiological mechanism that could, if substantiated, help explain the breadth of associations with oral breathing is the role of nasal nitric oxide (NO). The paranasal sinuses produce significant quantities of NO during nasal breathing, which is carried into the lower airways with each inhalation (32). Nasal NO serves multiple functions: it acts as a vasodilator that improves pulmonary oxygen uptake and systemic oxygen delivery, it has antimicrobial properties that contribute to innate immune defense in the upper and lower airways, and it plays a role in regulating ciliary motility for mucociliary clearance (7, 32). When breathing shifts from nasal to oral, this nasal NO contribution is largely bypassed; measurements in tracheotomized patients confirm that inhaled NO is predominantly of nasal origin (33). The loss of nasal NO may therefore contribute to both the dental and cognitive consequences of oral breathing through parallel mechanisms: reduced antimicrobial defense in the oral cavity may exacerbate susceptibility to caries, gingivitis, and periodontal disease, while reduced vasodilation may compromise cerebral oxygen delivery, contributing to the altered brain activity and local hemodynamic patterns observed in acute adult NIRS and fMRI studies (5, 25). No study to date has directly linked reduced nasal nitric oxide delivery to dentofacial abnormalities or to cognitive impairment in oral breathers. The pathway described here is therefore a biologically plausible but currently theoretical framework, offered to motivate future mechanistic research rather than to explain existing findings.

### Intervention and treatment evidence

While the majority of studies reviewed are observational, the question of whether resolving oral breathing reverses its consequences is clinically important. Several lines of evidence suggest that early intervention can improve outcomes. Adenotonsillectomy, the most common surgical treatment for airway obstruction in children, has been associated with improvements in sleep quality and behavioral outcomes in children with sleep-disordered breathing; notably, however, in the CHAT randomized trial it did not significantly improve the primary attention and executive-function outcome, despite improving behavior, quality of life, and polysomnographic findings (30). Studies on rapid maxillary expansion have reported improvements in nasal airway resistance and breathing patterns, with some evidence of subsequent improvements in sleep quality. Orthodontic interventions addressing narrow dental arches and malocclusion may also partially remediate craniofacial changes when initiated during growth periods. However, a critical limitation of the current evidence base is that no identified study has simultaneously measured both dental and cognitive outcomes before and after an intervention to restore nasal breathing. Such studies would provide the strongest test of the proposed mechanistic framework linking oral breathing to both domains. Future research should prioritize longitudinal, multi-outcome intervention designs that track dental, craniofacial, and cognitive changes following treatment of airway obstruction.

### Acute experimental versus chronic habitual oral breathing

The cognitive evidence reviewed here comprises two fundamentally different exposure models: acute, voluntary breathing-route manipulation over minutes to hours in healthy adults, and chronic, involuntary habitual oral breathing over months to years in children with airway obstruction. Acute experiments demonstrate that breathing route can modulate brain activity, local hemodynamics, and task performance in the moment; they do not demonstrate that chronic oral breathing produces cumulative neurodevelopmental harm. Conversely, chronic oral breathing in children is accompanied by sleep disruption, underlying airway disease, and possible adaptation, none of which is modeled in acute experiments. Findings from the acute adult paradigm are therefore treated in this review as mechanistic plausibility evidence only, and their clinical significance for children should not be overestimated.

### Confounding and alternative explanations

In many cases, oral breathing arises secondary to conditions such as adenotonsillar hypertrophy, allergic rhinitis, or other forms of upper airway obstruction, and these conditions may independently influence both craniofacial development and cognitive function, making it difficult to isolate the specific contribution of oral breathing itself. Sleep-disordered breathing and obstructive sleep apnea illustrate the difficulty: a systematic review of the pediatric OSA literature found that although intellectual ability in children with OSA may be impaired, it remains within the normal range, and that evidence for deficits in language, memory, attention, executive function, and academic performance is insufficient owing to low levels of evidence, conflicting findings, and heterogeneous measures (29) — an appraisal that argues for caution in the present review as well. Clinical guidance for childhood OSA emphasizes screening and referral rather than settled cognitive consequences (31), and in the CHAT randomized trial, adenotonsillectomy improved behavior, quality of life, and polysomnographic findings but did not significantly improve the primary attention and executive-function outcome (30). The associations observed in this review may therefore reflect a combination of effects of oral breathing and of its underlying causes and correlates. Future research should aim to better account for these factors, including study designs that distinguish between primary oral breathing and oral breathing secondary to identifiable medical conditions.

Among the pediatric studies contributing directly to the cognitive and academic synthesis, we did not identify standardized baseline intellectual assessment or statistical adjustment for baseline intellectual ability. Kuroishi et al. (23) applied controls at the design stage — enrolling only second- and third-year public elementary-school students, matching the groups for age and schooling, and excluding children reported to have psychological or neurological problems or to be receiving pedagogical or speech-therapy intervention — but administered no standardized measure of intellectual ability, and their group comparisons were unadjusted. Residual variation in IQ, visuospatial ability, executive function, and learning aptitude could therefore account for part of the observed group differences. Accordingly, the findings should not be interpreted as demonstrating that oral breathing causes reduced intellectual ability, and future studies should incorporate baseline cognitive assessment and adjust for it analytically.

Socioeconomic status, parental education, the home learning environment, and access to healthcare are well-established determinants of both cognitive development and academic achievement, and are also plausibly associated with exposure to untreated airway pathology, such as delayed diagnosis and treatment of adenotonsillar hypertrophy or allergic rhinitis. The pediatric studies reporting cognitive and academic outcomes in this review addressed these factors, at most, by design rather than by analysis: Kuroishi et al. (23) restricted enrollment to second- and third-year public elementary-school students and matched the groups for schooling, which restricts but does not eliminate socioeconomic variation — particularly as most of the mouth-breathing group was recruited from a university hospital clinic while controls were recruited from two local schools — and recorded socioenvironmental information during the clinical interview as an eligibility screen rather than as a statistical covariate. No reviewed study adjusted for socioeconomic status, parental education, or home learning environment in its analyses. Part of the reported association between oral breathing and academic or cognitive outcomes may therefore remain socioeconomically confounded.

Craniofacial morphology, airway dimensions, and facial growth patterns are all under substantial genetic influence. A child who inherits a narrow midface, a retrognathic mandible, or constricted nasal airways may be predisposed both to oral breathing and to the dentofacial phenotypes commonly attributed to it. Some of the associations summarized in this review may therefore reflect shared genetic determinants rather than a direct effect of breathing route on craniofacial development, a possibility that cross-sectional comparisons cannot exclude.

The studies reviewed here generally did not stratify results by sex, and most did not report sex-specific effects. Given known differences between males and females in craniofacial growth trajectories, airway anatomy, the prevalence of sleep-disordered breathing, and neurodevelopment, sex is a plausible modifier of both susceptibility to oral breathing and its consequences. The absence of sex-stratified data is a limitation of the current evidence base, and future studies should report and analyze outcomes by sex.

Finally, not all children who breathe orally develop malocclusion, craniofacial differences, or cognitive difficulties. Heterogeneity in outcomes may reflect differences in neuromuscular adaptation, tongue and lip posture, residual nasal airflow, age of onset, duration, severity, underlying airway pathology, genetic predisposition, or treatment history. We note that the direction of any such adaptation is not established: Masutomi et al. (6), included in this review, found lip-closing force and tongue pressure to be reduced rather than increased in mouth breathers, which does not support a straightforward compensatory account. These potential modifiers have not been adequately evaluated in the existing literature and should be regarded as hypotheses for future study. The group-level comparisons that dominate this field obscure such heterogeneity, and characterizing which individuals adapt successfully, and why, is an important direction for future research.

### Certainty of the conclusions

Because no formal risk-of-bias assessment was performed, certainty ratings such as GRADE were not assigned. The conclusions of this review are instead restricted to what the designs and recorded limitations of the included studies can support. For dentofacial outcomes, associations with oral breathing are reported by multiple observational studies, most of them cross-sectional, and a systematic review and meta-analysis that did apply formal appraisal rated the certainty of the cephalometric evidence as low to moderate (28); the present review supports the existence of these associations but not their direction, magnitude, or causal interpretation. For cognitive outcomes, the evidence consists of acute experiments in healthy adults with 9 to 36 participants per experiment and two cross-sectional pediatric studies of 55 and 30 children with unadjusted group comparisons (23, 34); these studies support the hypothesis that breathing route can modulate brain activity and task performance in the moment, but they do not establish that chronic oral breathing impairs cognition in children. For the proposed link between the dental and cognitive consequences of oral breathing, no included study examined both domains in the same participants, and the link remains a hypothesis. The limitations recorded in Table 1 point to biases acting in different directions: recruitment of mouth breathers from clinics and controls from the community, and the absence of adjustment for confounders, could inflate observed associations, whereas classification of breathing mode by parent report or questionnaire, if unrelated to the outcome, would tend to attenuate them. The net direction of bias cannot be determined without formal appraisal, and the associations summarized here should be interpreted with that uncertainty.

### Hypothetical framework linking dental and cognitive outcomes

While the findings presented in this review identify recurrent associations between oral breathing and both dental and cognitive outcomes, these relationships should be interpreted as correlational rather than causal. To generate testable hypotheses, a framework can be proposed that draws on the separate dental and cognitive literatures (Figure 3); it has not been tested in any single study.

**Figure 3:**
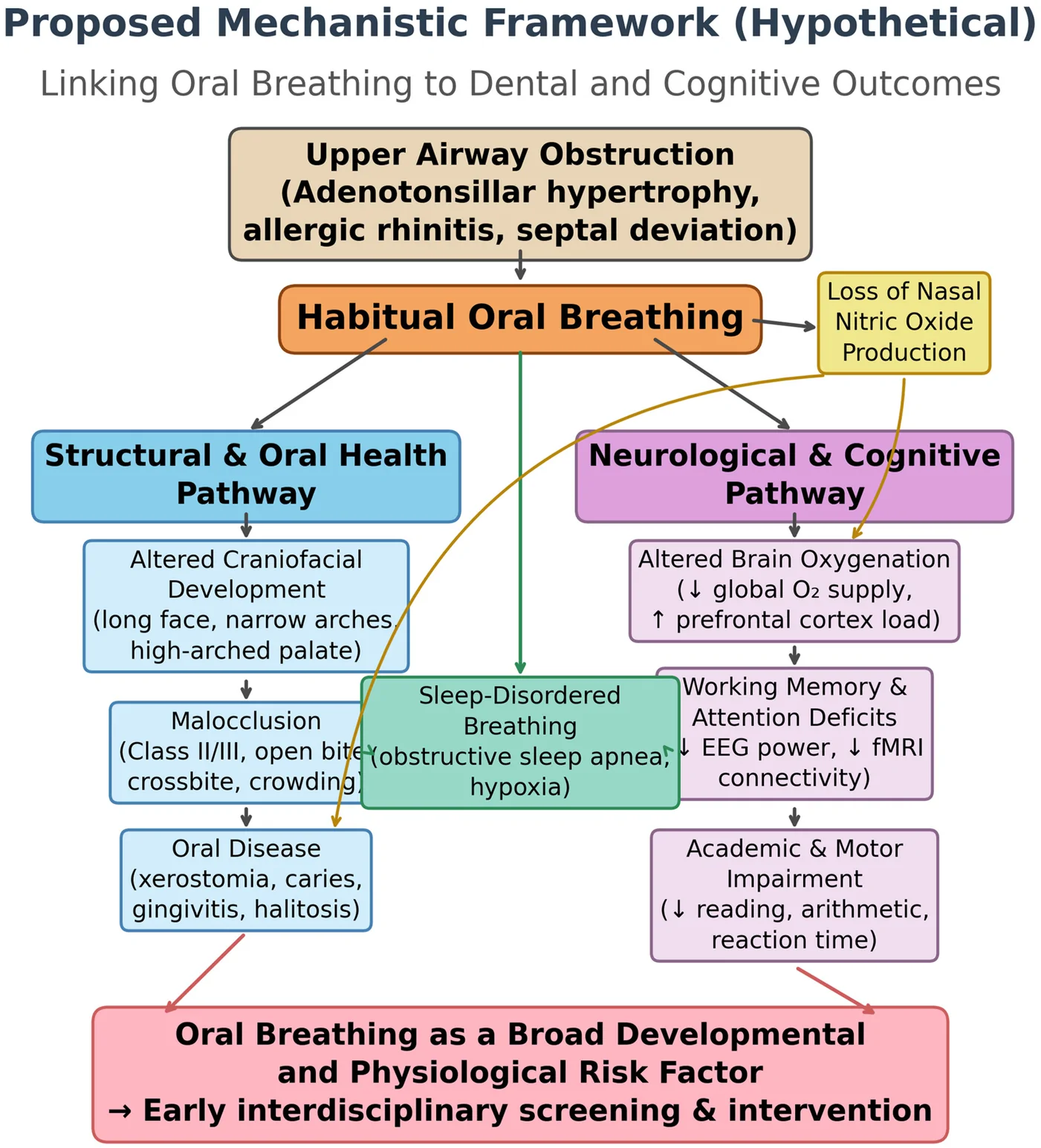
Hypothetical framework, not an established mechanistic pathway: proposed links between upper airway obstruction, habitual oral breathing, and parallel dental/craniofacial and neurological/cognitive outcomes. Loss of nasal nitric oxide production and sleep-disordered breathing are shown as hypothesized shared mediating pathways. Arrows represent hypothesized associations derived from the reviewed literature; none has been tested within a single experimental system.

Upper airway obstruction, such as adenotonsillar hypertrophy, may lead to habitual oral breathing. This altered breathing pattern may contribute to changes in craniofacial development, including malocclusion, altered jaw growth, and narrowing of the dental arches. At the same time, oral breathing may influence oxygen delivery and neural activity within the brain, potentially affecting cognitive processes such as memory, attention, and executive function. Loss of nasal nitric oxide delivery and sleep-disordered breathing are proposed as candidate shared pathways that could contribute to outcomes in both domains; neither has been examined as a mediator of both. If both sets of effects occur in the same children, they could produce co-occurring dental and cognitive outcomes; such co-occurrence has not been examined in the studies included in this review.

This proposed pathway is conceptualized as a sequence in which airway obstruction leads to oral breathing, followed by simultaneous effects on craniofacial development and brain function, ultimately resulting in downstream dental abnormalities and cognitive or behavioral consequences (Figure 3). While this framework provides a useful model for understanding the observed associations, it remains hypothetical and has not been directly tested within a single experimental system. Future research is needed to validate these relationships and clarify the underlying biological mechanisms.

### Clinical implications for pediatric dentistry

The findings of this review have important implications for pediatric dental practice. While oral breathing is often recognized for its effects on dentofacial development, its potential cognitive and behavioral associations highlight the need for a more comprehensive clinical approach. Dental professionals are in a unique position to identify early signs of oral breathing and its broader impacts during routine examinations.

Incorporating questions about sleep quality, snoring, daytime fatigue, attention, and school performance into patient history-taking may help identify children at risk for underlying airway or cognitive concerns. Observations such as malocclusion, narrow dental arches, and dry oral tissues, when paired with behavioral or academic difficulties, may warrant further evaluation. In such cases, referral to a pediatrician, otolaryngologist (ENT specialist), or sleep specialist may be appropriate to assess potential airway obstruction or sleep-disordered breathing.

Additionally, increasing awareness among parents about the potential link between oral breathing, sleep disturbances, and cognitive function can support earlier intervention. By recognizing oral breathing as a condition with both structural and functional implications, dental professionals can play a key role in interdisciplinary care aimed at improving both oral health and overall developmental outcomes in children.

A further consideration is the threshold at which oral breathing becomes clinically significant. The reviewed literature provides no dose–response data distinguishing mild, intermittent, or transient mouth breathing, such as that occurring during upper respiratory infections or seasonal allergy, from severe, persistent, habitual oral breathing, and no validated severity metric is in routine clinical use. It should not be assumed that all oral breathing carries equivalent developmental risk. The recognition and referral recommendations in this review are directed at persistent, habitual oral breathing, particularly when accompanied by structural signs, sleep symptoms, or behavioral and academic difficulties, and are not intended to pathologize transient mouth breathing. Defining clinically meaningful severity and duration thresholds is a priority for future research.

### Limitations

While this review aimed to comprehensively evaluate the associations between oral breathing and its dental and cognitive consequences, several limitations must be acknowledged. Only a single database (PubMed) was used for the primary searches, which may have introduced selection bias and restricted the breadth of the available literature. Although PubMed is a leading resource for medical and dental research, reliance on a single database may result in the underrepresentation of relevant studies, particularly within the cognitive and psychological literature that may be more extensively indexed in databases such as PsycINFO. Future reviews would benefit from incorporating additional databases, including Scopus, Embase, and Web of Science, to ensure a more comprehensive and representative evidence base. During revision, supplementary targeted searching outside PubMed identified one additional eligible study, which has been incorporated (34); this supplementary search was not a systematic multi-database search and does not establish complete coverage. The primary searches cover records indexed in PubMed up to 11 October 2024; studies indexed after that date were not screened.

The use of mutual exclusion terms in the two primary searches means that papers addressing both dental and cognitive outcomes of oral breathing simultaneously would be excluded from both search strategies. While a supplementary cross-domain search was conducted to partially mitigate this limitation, it cannot guarantee comprehensive capture of all relevant cross-domain literature.

The majority of included studies were observational or predominantly non-experimental in nature, limiting the ability to draw definitive causal inferences. As a result, the relationships described throughout this review should be interpreted as associative rather than causal. While a conceptual framework linking airway obstruction, oral breathing, craniofacial development, and cognitive outcomes can be proposed, this pathway has not been directly tested within a single experimental model. Longitudinal and interventional studies are needed to strengthen the evidence base and clarify the directionality of the observed associations.

In addition, although each included primary study has been classified by study-design level (OCEBM 2011) and annotated with its key limitations in Table 1, no formal risk-of-bias assessment was performed and no certainty ratings were assigned. The reasons are given in the Methods, and the consequences for each domain are set out under Certainty of the Conclusions; the conclusions of this review should be read as statements about the presence of associations and the plausibility of hypotheses, not about effect sizes or causal effects.

Variability across studies in diagnostic criteria for oral breathing, methods of assessing dental malocclusions and craniofacial development, and cognitive evaluation procedures further complicates direct comparisons. This heterogeneity may affect the generalizability of the findings and underscores the need for standardized definitions and methodologies in future research.

Several of the experimental studies informing the cognitive findings were conducted exclusively in adults under acute, voluntary manipulation of breathing route. Extrapolating such findings to the neurodevelopmental consequences of chronic oral breathing in children is a significant limitation of this synthesis, and pediatric conclusions drawn from that subset of studies should be regarded as hypothesis-generating rather than confirmatory.

Despite these limitations, this review brings together the dental and cognitive literatures on oral breathing and identifies testable hypotheses about their possible connection for future interdisciplinary investigation.

## Conclusions

This structured narrative review supports the view that habitual oral breathing is not a benign habit but a clinically relevant marker associated in multiple studies with dental and craniofacial differences, and, on a smaller and more preliminary evidence base, with differences in cognitive and academic performance. Multiple studies demonstrate associations between habitual oral breathing and malocclusion, altered craniofacial growth patterns, and increased vulnerability to oral disease. Emerging research also suggests associations with differences in memory, attention, and academic performance, with hypothesized contributions from altered brain oxygenation, loss of nasal nitric oxide signaling, and altered brain activity patterns, none of which has been tested directly.

These findings emphasize the importance of early detection, prevention, and interdisciplinary intervention strategies targeting oral breathing habits, particularly within pediatric populations. Clinical approaches that integrate dental, medical, and cognitive assessment may help identify children who warrant further evaluation; whether such approaches improve outcomes has not been tested. Future research should prioritize study designs that simultaneously assess both dental and cognitive outcomes within the same patient populations to better elucidate the relationship between these domains and strengthen evidence for shared underlying mechanisms.

### Clinical practice recommendations

The following recommendations for pediatric dental practice concern recognition and referral rather than treatment efficacy. Recommendations 1, 2, and 4 are informed indirectly by existing pediatric guidance on the evaluation of sleep-disordered breathing, which recommends that all children be screened for snoring and that symptomatic children be referred for further assessment (31). Recommendations 3, 5, and 6 represent expert-informed clinical considerations. Direct evidence that implementation of these recommendations improves dental or cognitive outcomes in children with oral breathing is currently lacking.

1. Incorporate airway and breathing assessments into routine examinations, including observation of mouth breathing, lip incompetence, and dry oral tissues.
2. Include targeted questions in patient history-taking, such as inquiries about snoring, sleep quality, daytime fatigue, attention, and school performance.
3. Recognize combined dental and behavioral indicators, where signs such as malocclusion or narrow dental arches occur alongside attention or learning difficulties, as a reason for further evaluation; this combination has not been validated as a predictor of airway or cognitive problems.
4. Provide early referrals when appropriate, particularly to pediatricians, otolaryngologists (ENT specialists), or sleep specialists for evaluation of potential airway obstruction or sleep-disordered breathing.
5. Educate parents on the broader implications of oral breathing, including its potential impact on both oral health and cognitive development, to encourage early intervention and compliance with treatment recommendations.
6. Adopt an interdisciplinary approach to care, collaborating with medical and behavioral health professionals to address both structural and functional aspects of oral breathing.
