# Supplementary material for: Oral breathing and its dental and cognitive implications: a structured narrative review with a pediatric focus
Source: Front Dent Med. 2026 Oct 27;7:1868240. doi: 10.3389/fdmed.2026.1868240 (PMC13748960; doi:10.3389/fdmed.2026.1868240)
Supplement: Supplementary file 1 [file Supplementaryfile1.docx]

*Oral breathing and its dental and cognitive implications: a structured narrative review with a pediatric focus*

Alex Towery and Gopikrishna Deshpande

**Supplementary Table S1. Source of each included study.**

| **Ref** | **Study** | **Identified through** |
| --- | --- | --- |
| [1] | Lin et al., 2022* | PubMed Search #1 |
| [2] | Farid & Metwalli, 2010 | PubMed Search #2 |
| [3] | Alhazmi, 2022 | PubMed Search #1 |
| [4] | Jefferson, 2010* | PubMed cross-domain search |
| [5] | Jung & Kang, 2021 | PubMed cross-domain search |
| [6] | Masutomi et al., 2024 | PubMed cross-domain search |
| [7] | Tamkin, 2020* | PubMed cross-domain search |
| [8] | Grippaudo et al., 2016 | PubMed Search #1 |
| [9] | Jaiswal et al., 2023 | PubMed Search #1 |
| [10] | Lee et al., 2021 | PubMed Search #1 |
| [11] | Paolantonio et al., 2019 | PubMed Search #1 |
| [12] | Huynh et al., 2014* | PubMed Search #1 |
| [13] | Lysy et al., 2021 | PubMed Search #1 |
| [14] | Nagaiwa et al., 2016 | PubMed Search #1 |
| [15] | Wang et al., 2023 | PubMed Search #1 |
| [16] | Bakor et al., 2010 | PubMed Search #1 |
| [17] | Kaur et al., 2018 | PubMed Search #1 |
| [18] | Alqutami et al., 2019 | PubMed Search #1 |
| [19] | Jiménez et al., 2017 | PubMed Search #1 |
| [20] | Shahsavar et al., 2024* | PubMed Search #2 |
| [21] | Arshamian et al., 2018 | PubMed Search #2 |
| [22] | Lee et al., 2020 | PubMed Search #2 |
| [23] | Kuroishi et al., 2015 | PubMed Search #2 |
| [24] | Hong et al., 2021 | PubMed Search #2 |
| [25] | Sano et al., 2013 | PubMed Search #2 |
| [26] | Yoshimura et al., 2019 | PubMed Search #2 |
| [27] | Jung et al., 2020 | PubMed Search #2 |
| [34] | Braga Junior et al., 2020 | Supplementary Semantic Scholar search, 27 August 2026 |

* Review or editorial used as contextual background only. PubMed searches were run in October 2024; restricting each string to records entered in PubMed on or before 11 October 2024 reproduces the reported counts (Search #1, 246; Search #2, 117; cross-domain search, 92).
